# MR Lymphangiography: A Practical Guide to Perform It and a Brief Review of the Literature from a Technical Point of View

**DOI:** 10.1155/2017/2598358

**Published:** 2017-03-07

**Authors:** Francesco Giuseppe Mazzei, Francesco Gentili, Susanna Guerrini, Nevada Cioffi Squitieri, Duccio Guerrieri, Paolo Gennaro, Michele Scialpi, Luca Volterrani, Maria Antonietta Mazzei

**Affiliations:** ^1^Diagnostic Imaging, Azienda Ospedaliera Universitaria Senese, Viale Bracci 10, 53100 Siena, Italy; ^2^Department of Medical, Surgical and Neuro Sciences, Diagnostic Imaging, University of Siena, Azienda Ospedaliera Universitaria Senese, Viale Bracci 10, 53100 Siena, Italy; ^3^Department of Maxillofacial Surgery, University of Siena, Viale Bracci 10, 53100 Siena, Italy; ^4^Department of Surgical and Biomedical Sciences, Division of Radiology 2, Perugia University, S. Maria Della Misericordia Hospital, Perugia, Italy

## Abstract

We propose a practical approach for performing high-resolution MR lymphangiography (MRL). We shall discuss and illustrate the technical approach for the visualization of lymphatic vessels in patients suffering from lymphedema, how to distinguish lymphatic vessels from veins, and MRL role in supermicrosurgery treatment planning. A brief review of literature, from a technical point of view, is also reported.

## 1. Introduction

**Figure 1:**
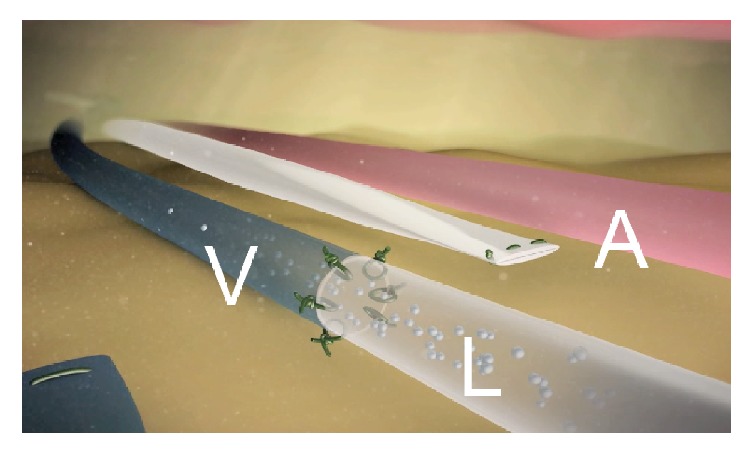
Depiction of end-to-end lymphaticovenous anastomosis (LVA) to treat lymphedema; V = vein, L = lymphatic vessel, and A = excluded lymphatic vessel.

Lymphedema is the result of a compromised lymphatic drainage caused by injury to the lymphatics followed by an exaggerated accumulation of lymphatic fluid in the interstitial tissue [1]. Today, the implementation of microsurgical lymphovenous shunts (supermicrosurgical treatment), planned to achieve a natural outflow steering lymphatic flow to the venous system overcoming the site of the lymphatic obstruction, is the preferred method for the treatment of lymphedema [2] (Figure 1). In this scenario, Magnetic Resonance Lymphangiography (MRL), combining morphological and functional information in a single examination, could play a pivotal role in treatment planning. In particular the entire lower or upper extremity can be examined in several steps with high spatial and temporal resolution, obtaining dynamic information of contrast agent uptake of both lymph nodes and lymphatic vessels [3]. Thanks to the detailed anatomical information regarding the lymphatic system, MRL could also be useful in evaluating changes in the lymphatic circulation postoperatively or in the event of surgical complications [4]. This article illustrates the MRL technical approach for imaging lymphatic vessels in patients with lymphedema, how to distinguish lymphatic vessels from veins, and MRL use in planning lymphaticovenous anastomosis (LVA) treatment. A brief review of literature, from a technical point of view, is also reported.

## 2. Case History

From February 2014 to September 2016 we enrolled 30 patients (24 women) with a mean age of 30 years (range 18–70); all of them underwent LVA intervention within 72 hours after MRL examination; 17 out of 30 were affected by lower limb lymphedema with 6 cases of primary lymphedema; the others were secondary to cancer treatment. All procedures performed in this study involving human participants were undertaken in accordance with the ethical standards of the institutional and/or national research committee and with the 1964 Helsinki Declaration and its later amendments or comparable ethical standards. Informed consent was obtained from all individual participants included in the study.

## 3. General Technique of High-Resolution MR Lymphangiography

The MRL technique could vary slightly depending on the MR equipment and the anatomical site of investigation but can be outlined as follows.

### 3.1. MR Equipment

The preferred MR equipment includes a 1.5-Tesla or more MR unit. In our experience, all MR examinations were performed by a General Electric Healthcare Signa TwinSpeed HDxt, with a maximum gradient strength value of 23 mT/m and a slew rate of 80 mT/m/ms (software release 15.0_0947A). A multielement body coil is fundamental for this type of examination. For our purposes we used a receiving phased-array peripheral vascular coil for the study of the lower extremities (Flow 7000 phased-array peripheral vascular, USA Instruments) and an 8-channel body array coil for the upper extremities, with both a large anatomical coverage and a good signal-to-noise ratio.

### 3.2. Positioning of the Patient

Patients should be fully informed about the procedure to confirm their complete collaboration. Positioning varies depending on the anatomical site of investigation.

**Figure 2:**
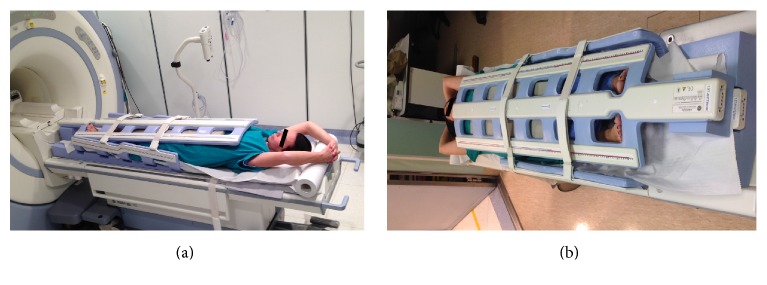
Patient's position for the study of the lower limb.

*(i) Lower Limb*. Patient is placed in the supine position, feet first, with both legs on a ramp pillow so that the lower extremity is parallel to the main magnetic field and near the most homogeneous area of B0. According to the height of the patient, three or four stations are examined in order to cover the following anatomical regions: (1) the lower leg inferior segment and foot region (feet region); (2) the lower leg superior segment and upper leg inferior segment, including knee region (calf region); (3) the middle upper leg and the proximal upper leg including inguinal region (thigh region and pelvic region). The toes of both feet emerge from the holes of the coil and are easily accessible for the injection of the contrast agent (Figure 2).

**Figure 3:**
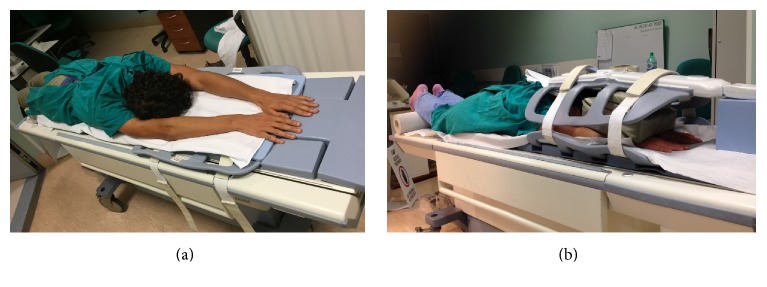
Patient's position for the study of the upper limb.

*(ii) Upper Limb*. The same procedure is used to study the upper extremity but the patient is in the prone position, head first (Figure 3). Two stations are usually examined in order to cover the following anatomical regions: (1) hand-wrist-forearm and (2) elbow-arm-shoulder (axilla). Direct contact of the coil with the skin must be avoided by means of small cushions to reduce the hyperintensity artifacts.

### 3.3. Insertion of the Needle

**Figure 4:**
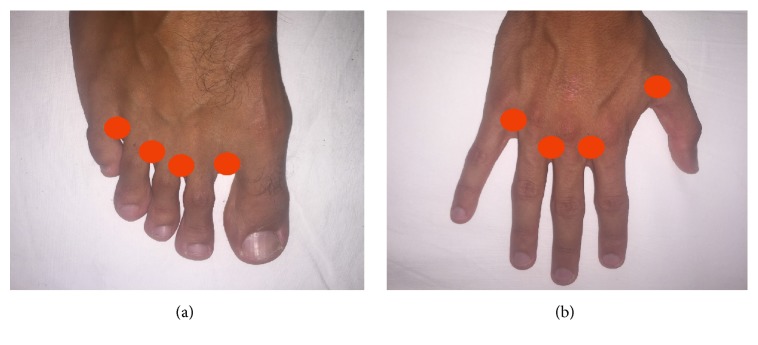
Sites of injection of the contrast media.

A 24–28-Gauge (G) thin needle is generally preferred. Ideally, the tip of the needle should gently be inserted subcutaneously into the dorsal aspect of each foot or hand in the region of the four interdigital web spaces (Figure 4). The injection is limited to a maximum volume of 2 mL (generally 1 ml) for each interdigital web space.

### 3.4. Contrast Agent Administration

A mixture of the standard dose (0.1 mmol/kg body weight) of a paramagnetic contrast medium and 0.5 mL of lidocaine 1% for local anaesthesia is injected subcutaneously/intradermally. For our purposes, the contrast agent used was gadobenate dimeglumine (Gd-BOPTA, Multihance, Bracco Imaging, Milan, Italy). Since experimental animal models have only shown minor tissue damage after intracutaneous injection or extravasation, a gadolinium agent offers an acceptable safety profile for intracutaneous administration at the recommended dose, even if it is still considered as an off-label use [5–8]. Lidocaine 1% is administered with the contrast medium also to alleviate pain during the injection. Generally no complications are observed after the examination, in particular during or after intracutaneous injection of Gd-BOPTA.

### 3.5. MR Parameters and Sequences

The imaging protocol generally consists of a heavily T2-weighted sequence in order to evaluate the extent and distribution of the lymphedema and of a 3D fast spoiled gradient-echo T1-weighted sequence with a fat-saturation technique for the lymphatic visualization [3, 9, 10]. In our experience we performed a 3D steady-state free precession (SSFP) balanced electrocardiography- (ECG-) triggered sequence (FIESTA, GE) with spectral fat saturation (SPECtral inversion at lipid, SPECIAL, GE) instead of a heavily T2-weighted sequence in order to obtain a good visualization of both the venous system and the distribution of the lymphedema within the same sequence and at the same time. The study was conducted in three steps: (1) a survey and a mandatory calibration were performed for all stations, three or four for the lower extremity (foot-ankle-calf, calf-knee, and thigh-hip) and two or three for the upper extremity (hand-wrist-forearm, elbow-arm-shoulder). Before injection of the contrast medium, a coronal 3D SSFP-balanced ECG-triggered sequence with spectral fat saturation (SPECtral inversion at lipid, SPECIAL, GE) was acquired. The ECG-trigger was acquired with a peripheral gating (PG, GE) and a time delay is set for a systolic phase acquisition in order to obtain non-contrast-enhanced venograms and clear images for the visualization of lymphedema. We then performed a precontrast coronal 3D spoiled gradient-recalled echo T1-weighted sequence with SPECtral inversion at lipid (FSPGR with SPECIAL, GE) in all stations in order to increase contrast sensitivity and then subtracted this precontrast sequence (“mask”) from subsequent postcontrast images; (2) the patient is brought out of the bore and instructed not to move. Two radiologists begin to inject the contrast medium simultaneously (one for each extremity), using a 28G thin needle inserted consecutively into the dorsal interdigital spaces of both the extremities; (3) the first station is repeated 5, 20, and 35 minutes after the injection of the contrast medium. The other one/two stations are examined in sequence after the first station at each fixed time (5, 20, and 35 minutes). Each 3D SSFP-balanced sequence lasts about 3 minutes and each 3D spoiled gradient-recalled echo T1-weighted sequence lasts nearly 3 minutes and 50 seconds, with a total average examination time of 1 hour and 15 minutes for the lower limb (3 minutes × 3/4 anatomical regions/stations and 3 minutes and 50 seconds × 3/4 anatomical regions/stations × 4 times [time of 0, 5, 20, and 35 minutes]) and 50 minutes for the upper limb. The technical parameters used for the suggested sequences are shown in Table 1.

## 4. Image Analysis

The source images of each sequence should be reviewed on a 3D workstation to allow for the real-time creation of rotating 360° 3D postprocessed images. Multiplanar reformations (MPR), thin-section maximum intensity projection (MIP) reconstructions (section thickness 10–15 mm), and the 3D pointer should be used to identify and localise the different lymphatic and vascular structures. A long-extremity display composed of all two-four anatomical stations should be generated using dedicated software. The postprocessed images, with the essential spatial and depth information, should then be recorded in the picture archiving and communication system (PACS), so that they are easily accessible to the surgeon before performing LVA.

### 4.1. Characterisation of Lymphatic Vessels

**Figure 5:**
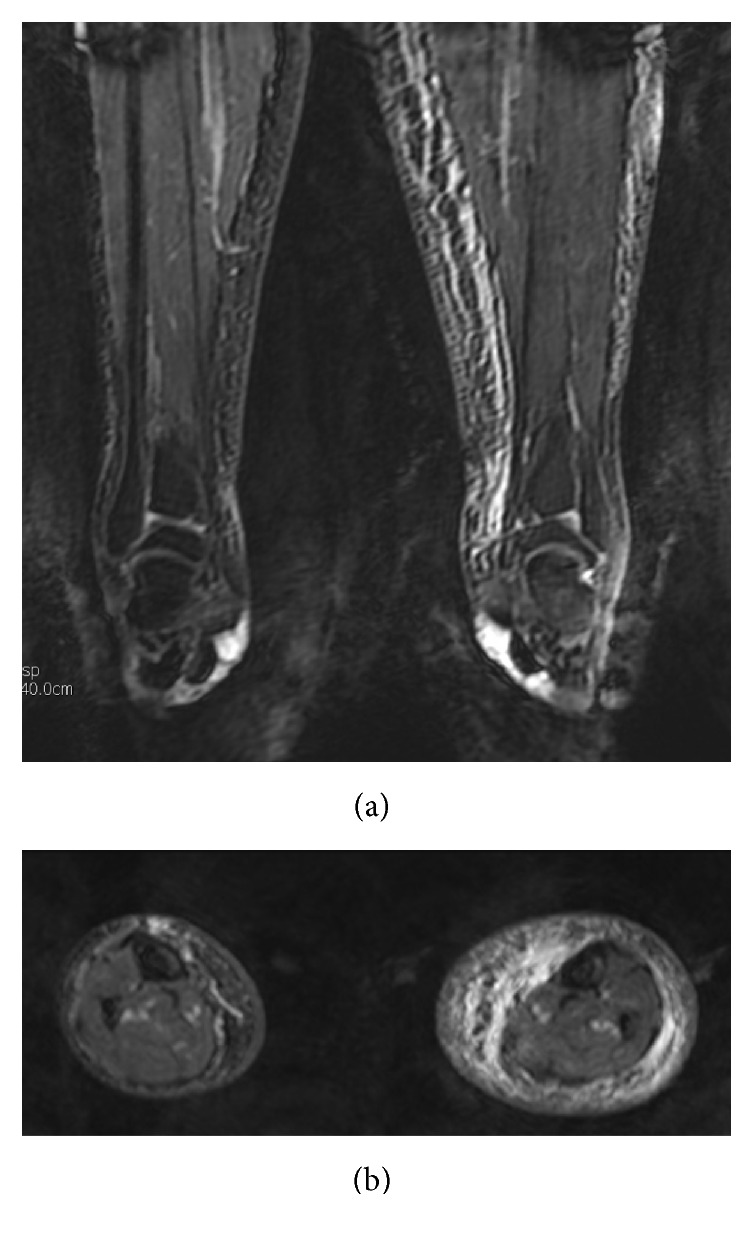
Coronal and axial 3D SSFP-balanced MIP images depict the characteristic muscle-sparing epifascial distribution of lymphedema.

**Figure 6:**
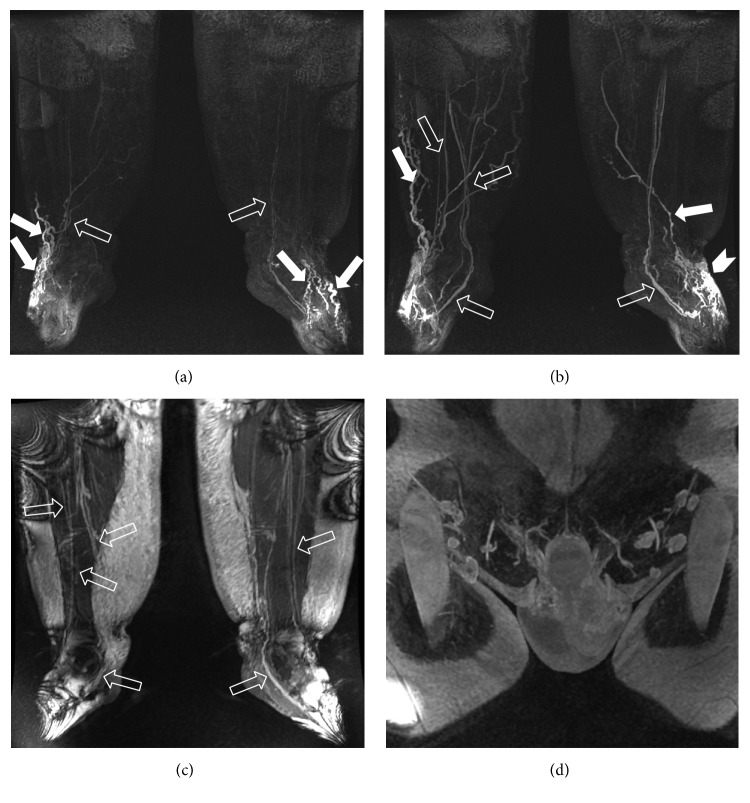
MRL (1,5 T, GE) in a 43-year-old man with congenital primary lymphedema. 3D frontal spoiled gradient-echo MIP after 5 (a) and 20 (b) minutes show a progressive delineation and enhancement of lymphatic vessels (white solid arrows) with an extensive area of dermal backflow (interstitial dispersion of the contrast medium in soft tissue due to proximal obstruction of lymph drainage) in the left foot (arrow head in (b)); please note the beaded appearance of lymphatics comparing to the substantially more rectilinear shape of veins (open arrows). The possibility of visualizing a precontrast venogram through a 3D steady-state free precession (SSFP) balanced sequence makes the distinction between veins and lymphatic vessels easier. The optimal depiction of the high-intensity epifascial lymphedema (c) and inguinal lymph nodes (d) is also evident.

Concomitant venous contamination is generally detected in each exam, as reported extensively in previous works using gadolinium-based contrast agent [5, 11, 12]. The lymphedema shows an epifascial distribution with a high-signal intensity in coronal 3D SSFP-balanced images (Figure 5). Pathological lymphatic vessels are usually clearly visible and recognised by their tortuous and beaded appearance, whereas the adjacent veins are straight with focal bulging only in the vicinity of venous valves. Other aspects, often associated with lymphatics, include dermal backflow (an area of progressive interstitial dispersion of the contrast medium in soft tissue due to proximal obstruction of lymph drainage) and collateral transport pathways (honeycombing); these characteristics are visible after a mean time of 15–20 minutes from the injection of the contrast media, and their intensity increased over time (Figure 6). The mean maximum diameter of affected lymphatic vessels is similar to that of adjacent veins but greater than lymphatic vessels in the healthy limb, the latter rarely visualized. In fact, under normal conditions, in a healthy lymphatic system the lumen of the vessel is almost virtual [9]. In addition, another feature that can help to differentiate lymphatics from adjacent veins is the kinetic of the enhancement, and in fact lymphatic vessels and veins show different enhancement times and different times to peak enhancement. In particular, despite the almost simultaneous initial enhancement of both veins and lymphatic vessels, after 5–10 minutes from the injection of the contrast agent, because of the continuous higher flow, veins wash-out occurs in later sequences while affected lymphatic vessels remain enhanced, presumably due to lymph stasis.

### 4.2. How to Plan LVA Treatment: MR Report

After reviewing and postprocessing of the images, a proper MRL report should include the following data:

1. The presence, severity (extension and thickening), and location of the lymphedema.
2. The number, diameter, course, and depth from the skin of both affected lymphatic vessels and the nearest veins.
3. The exact distance between the affected lymphatic vessel and the vein chosen for the LVA.
4. The lymphatic drainage pattern (type 1: poor lymphatic drainage or diffuse interstitial enhancement known as dermal backflow; type 2: partially diffuse enhancement or interstitial and vascular enhancement, if some lymphatic vessels are depicted in the area of the dermal backflow (honeycombing); type 3: directed, if there is lymphatic enhancement without the dermal backflow).
5. The delay of drainage (score 0: no drainage; score 1: substantial delay [pelvic or axilla level >60 minutes or not reached before the end of the examination]; score 2: slight delay [pelvic or axilla level >20 minutes]; score 3: no delay [lymphatic vessels enhancement obtained in the first series of images or reached pelvic or axilla level <20minutes]).
6. The detection and localisation of lymph nodes.
7. The presence of venous contamination (present or not present) and whether it compromises the diagnosis and the presence of lymphangiectasia (yes or no) should be also reported.

## 5. Discussion

**Figure 7:**
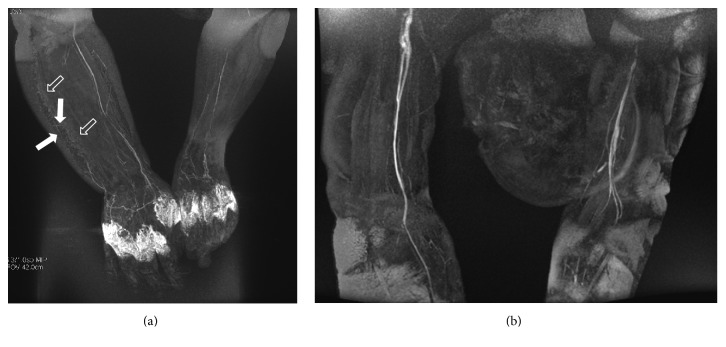
MRL in a 52-year-old woman with high-grade upper right limb lymphedema secondary to lymphadenectomy for a breast cancer. 3D frontal spoiled gradient-echo MIP after 35 minutes from the contrast agent administration (a) shows only some discontinuous lightly enhanced skin lymphatic vessels (white solid arrows) in the inferior lateral portion of the affected limb within a honeycombing area (open arrows). No pathological lymphatic vessels are seen in the upper right arm after 45 minutes.

**Figure 8:**
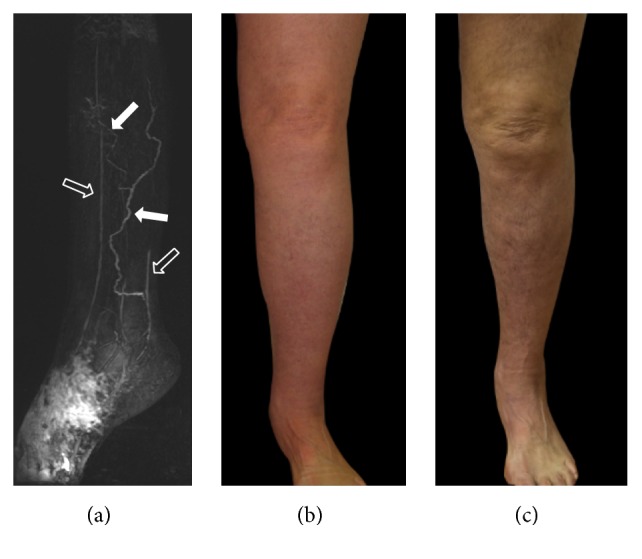
MRL and clinical appearance, before (a, b) and after LVA (c), of the left lower limb in a 67-year-old woman with unilateral lymphedema secondary to a pelvic carcinoma. MRL (a) depicts pathological lymphatic vessels (white solid arrows) and adjacent veins (open arrows) to perform the anastomoses. Changes in limb diameter and skin colour are clear two months after treatment (c).

Lymphedema is a chronic debilitating condition that is frequently misdiagnosed and traditionally regarded as incurable [3, 9, 13]. It results from impaired lymphatic transport caused by damage to lymphatic vessels, infection, or congenital abnormality [14, 15]. In our clinical experience, lymphedema is due to malignancy or cancer therapy in a majority of patients and to breast cancer surgery in about 50% of cases. LVA, a surgical treatment where collecting lymphatic vessels are anastomosed to a cutaneous vein under surgical microscopy, has been demonstrated to improve lymphatic drainage, reduce limb diameter, and avoid dermal sclerosis. It is the current preferred surgical treatment for this pathological condition [16]. An alternative microvascular surgical technique is represented by lymph node transfer, which means moving normal lymph nodes and associated adipose tissue to the anatomical region of the body affected by lymphedema [17]. Prior to supermicrosurgery treatments, these patients need to undergo appropriate imaging for distinguishing lymphatic vessels from veins and their anatomical position in order to plan the best strategy for microsurgical lymphatic vessel reconstruction. Compared to radioisotope lymphoscintigraphy which could have a role in demonstrating the dermal backflow and lymph node drainage but which is limiting in the visualization of lymphatic vessels due to its lower spatial resolution, MRL is a promising technique for supplying more accurate functional and anatomical information due to its better spatial and temporal resolution, depicting the drainage pattern, lymph node position, lymphatics, and venous structures, as well as the severity of lymphedema [12, 18, 19]. Moreover this technique is minimally invasive due to the lack of ionising radiation and good tolerability of subcutaneous injection by patients. Some limitations of MRL must be highlighted: the long duration of the MR examination and the occasional difficulty in distinguishing the affected lymphatic vessels when an underlying remarkable venous contamination is present. In fact while the colloid-binding tracer of lymphoscintigraphy is very specific for the lymphatic system, gadolinium chelates are water soluble and diffusible, so that venous drainage of the contrast agent may also be present. Regarding this limitation, despite White et al. reporting the need of an intradermal injection rather than a subcutaneous injection for the optimal visualization of lymphatics and poor venous contamination [3], we did not find significant differences between the two approaches. In our experience, the only precaution adopted before the contrast medium injection was to withdraw the syringe plunger in order to avoid a small vein cannulation. From a strictly technical point of view, even if some authors [20] still claimed that noncontrast MR lymphangiography using very heavily T2-weighted Fast Spin-Echo (FSE) sequences is a unique, non invasive, imaging modality for the diagnosis of lymphedema, the majority of authors perform MRL using both heavily T2-weighted and heavily T1-weighted postcontrast sequences. In particular Lu et al. compared heavily T2-weighted with 3D fast spoiled gradient-recalled echo T1-weighted sequences, reporting a high possibility of identifying with the former not only lymphedema but also lymphatic vessels, despite some difficulties in distinguishing diffuse subcutaneous infiltration with a honeycombing pattern from small lymphatics. In addition, they suggest performing both sequences for an optimal examination [9, 21]. Recently Jeon and colleagues compared 3T contrast 3D isotropic T1-weighted FSE and contrast 3D isotropic intermediate-weighted FSE sequences and claimed that 3D isotropic T1-weighted FSE provides better information regarding lymphatic vessels, whereas lymph node detection is lower. Conversely 3D isotropic intermediate-weighted FSE sequence has the advantage of depicting lymph nodes in lymphedematous extremities but demonstrating a lower detection of lymphatic vessels. In fact as the intermediate-weighted FSE sequence reflected the T2 effect using a driven pulse, subcutaneous oedema and slow-flow structures, such as the venous system, could also be seen together with the lymphatic vessels [22]. To overcome this limitation and since intracutaneously administered contrast agent is simultaneously absorbed by the venous circulation Mitsumori and colleagues, after 3D heavily T2-weighted sequence to depict the severity of lymphedema and a high-resolution fat suppressed 3D spoiled gradient-echo (3D-SPGR) sequence after the intracutaneous injection of Gd-based MR contrast to image lymphatic vessels, concluded the examination with an intravenous injection of Gd-based MR contrast to obtain an MR venogram by repeating the high-resolution 3D SPGR sequence, using the images from the MR venogram to facilitate the differentiation of superficial veins from enhancing lymphatic vessels during exam interpretation. On the contrary we prefer to perform a 3D SSFP balanced sequence instead of a heavily T2-weighted sequence before 3D gradient-echo T1-weighted MRL, to obtain at the same time the depiction of the severity and distribution of lymphedema and a visualization of a precontrast venogram, thus facilitating the subsequent distinction between veins and lymphatic vessels and also reducing the examination time [23]. Furthermore, we would like to point out the importance of a precontrast sequence in performing 3D MRL, in order to subtract it from the later postcontrast images. In fact, although Mitsumori et al. did not find this technique useful, as it was invalidated by patient movements [10], we found an advantage from this approach in the visualization of small lymphatic vessels; evidently the patient should be instructed to maintain complete collaboration. In our experience only in 3 out of 24 patients with secondary lymphedema, we observed a poor lymphatic drainage limited to the lower part of the limb, because of the extremely impaired lymphatic circulation (Figure 7); therefore in these cases LVA treatment was restricted to this anatomical region. After surgery a clinical improvement was observed in all patients within 1-2 months (Figure 8) without significant complications, so MRL follow-up was not required.

## 6. Conclusions

MRL with gadolinium contrast agent is a minimally invasive and safe technique. It provides good morphological and functional information in a single examination and represents the current best method for planning an optimal surgical treatment for patients suffering from lymphedema. In this pictorial review we described the most common techniques used to perform MRL, in order to offer practical guidance for achieving high-quality MRL images.

## Figures and Tables

**Table 1:** Imaging parameters for magnetic resonance lymphangiography at 1,5 T.

	TR	TE	TI	FA (°)	FOV (cm)	Matrix	Thickness/overlap (mm)	NEX	Bandwidth (khz)
Coronal 3D SSFP balanced^*∗*^	*4.0*	*1.9*	*90*		*40 × 40*	*224 × 192*	*2/1*	*0.53*	*±125*
Coronal 3D spoiled GRE T1W with SPECtral inversion at lipid balanced^*∗*^	*5.0*	*2.1*	*17*	*25*	*44 × 44*	*448 × 320*	*2.8/1.4*	*1*	*±111.1*
3D T2-weighted turbo spin-echo	2000	680			40 × 40	320 × 224	3.5/1	1	*±31.2*

TR = repetition time; TE = echo time: TI = inversion time; FA = flip angle; FOV = field of view; NEX = number of excitations.

^*∗*^Sequences performed in our experience.
